# Seasonal and annual fluctuations of deer populations estimated by a Bayesian state–space model

**DOI:** 10.1371/journal.pone.0225872

**Published:** 2020-06-18

**Authors:** Inoue Mizuki, Hiroki Itô, Michimasa Yamasaki, Shigeru Fukumoto, Yuuki Okamoto, Masaya Katsuki, Keitaro Fukushima, Masaru Sakai, Shota Sakaguchi, Daisuke Fujiki, Hikaru Nakagawa, Masae Iwamoto Ishihara, Atsushi Takayanagi

**Affiliations:** 1 Department of Biosciences, College of Humanities and Sciences, Nihon University, Tokyo, Japan; 2 Hokkaido Research Center, Forestry and Forest Products Research Institute, Sapporo, Hokkaido, Japan; 3 Laboratory of Forest Biology, Graduate School of Agriculture, Kyoto University, Kyoto, Japan; 4 General Office in Hirano-cho, Osaka, Japan; 5 Center for Ecological Research, Kyoto University, Otsu, Shiga, Japan; 6 Fukushima Branch, National Institute for Environmental Studies, Tsukuba, Japan; 7 Graduate School of Global Environmental Studies, Kyoto University, Kyoto, Japan; 8 Institute of Natural and Environment Sciences, University of Hyogo, Sanda, Hyogo, Japan; 9 Center for Southeast Asian Studies, Kyoto University, Kyoto, Japan; 10 Field Science Education and Research Center, Kyoto University, Kyoto, Japan; Nanjing Forestry University, CHINA

## Abstract

Deer overabundance is a contributing factor in the degradation of plant communities and ecosystems worldwide. The management and conservation of the deer-affected ecosystems requires us to urgently grasp deer population trends and to identify the factors that affect them. In this study, we developed a Bayesian state–space model to estimate the population dynamics of sika deer (*Cervus nippon*) in a cool-temperate forest in Japan, where wolves (*Canis lupus hodophilax*) are extinct. The model was based on field data collected from block count surveys, road count surveys by vehicles, mortality surveys during the winter, and nuisance control for 12 years (2007–2018). We clarified the seasonal and annual fluctuation of the deer population. We found a peak of deer abundance (2010) over 12 years. In 2011 the estimated deer abundance decreased drastically and has remained at a low level then. The deer abundance gradually increased from April to December during 2013–2018. The seasonal fluctuation we detected could reflect the seasonal migration pattern of deer and the population recruitment through fawn births in early summer. In our model, snowfall accumulation, which can be a lethal factor for deer, may have slightly affected their mortality during the winter. Although we could not detect a direct effect of snow on population dynamics, snowfall decrease due to global warming may decelerate the winter migration of deer; subsequently, deer staying on-site may intensively forage evergreen perennial plants during the winter season. The nuisance control affected population dynamics. Even in wildlife protection areas and national parks where hunting is regulated, nuisance control could be effective in buffering the effect of deer browsing on forest ecosystems.

## Introduction

In the past few decades, deer have become increasingly abundant worldwide [1, 2]; this population increases have contributed to the degradation of plant communities and ecosystems [3–6]. In general, the population dynamics of animals are affected by birth, mortality, and migration rates. Large ungulates are able to breed under low food availability [7], therefore, the birth rate of deer would not largely decrease even in a degraded forest; however, the density-dependent decline in the birth rate of deer occurs at a later period of the outbreak stage [8]. Furthermore, the survival rate of adult deer was high even in a poor nutritional environment [9]. Thus, deer is a species that can live in high densities and low-nutrient environments. If predators (e.g. wolves) are absent, hunting is one options to control deer populations under these conditions [10, 11].

In snow-covered area and, in particular, during heavy snowfall, the survival rate of sika deer (*Cervus nippon*) decreases [9, 12]. Kawase et al. (2014) [13] projected that winter precipitation including snowfall would decrease in broad regions of Japan due to the ongoing climate change. This climate change may mitigate the mortality of deer and cause further increases in deer populations in the future. Therefore, it is indispensable to estimate the effect of snow on the dynamics of deer populations. While some of the effects of global warming on population dynamics of ungulates have already been reported [14–16], models constructed in recent studies to describe deer population dynamics have not yet explicitly considered the effects of snow.

From the viewpoint of plant communities and ecosystems, it is important to clarify not only annual trends but also seasonal trends in the deer population density. Plant fitness could be affected differently depending on whether deer browse on them before or after they have reproduced sexually. The timing of browsing could also affect the fitness of pollinators such as bumblebees. Therefore, in order to assess the effects of deer browsing on ecosystem levels it is important to, at least, estimate the seasonal deer abundance. However, in many areas, the annual census of ungulates is held during a season that, although offers good visibility to track ungulates, is not suitable for plant growth [10, 15, 17].

In recent years, generalized linear models [18], generalized additive mixed models (GAMM, [19]), density surface models [20], and Bayesian state-space models [10, 16] were used to estimate deer abundance based on field data. Among these models, the Bayesian state-space model can be a powerful tool for estimating deer population dynamics because it can easily handle time series data with temporal autocorrelation and can explicitly distinguish errors following measurement of data with uncertainty about population dynamics [10, 21]. However, there are still limited applications of this model when it comes to the effect of snow and seasonal fluctuations on deer population dynamics.

In this study, we estimated deer population dynamics in a cool-temperate forest in Japan using a Bayesian state-space model. The model was based on data collected from block count surveys, road count surveys by vehicles, mortality surveys during the winter, and nuisance control over 12 years. The seasonal and annual fluctuation of the sika deer population and the effects of snowfall and nuisance control on population dynamics are discussed based on the results we obtained by the model and the parameters estimated in the model, respectively.

## Materials and methods

### Study site

**Fig 1:**
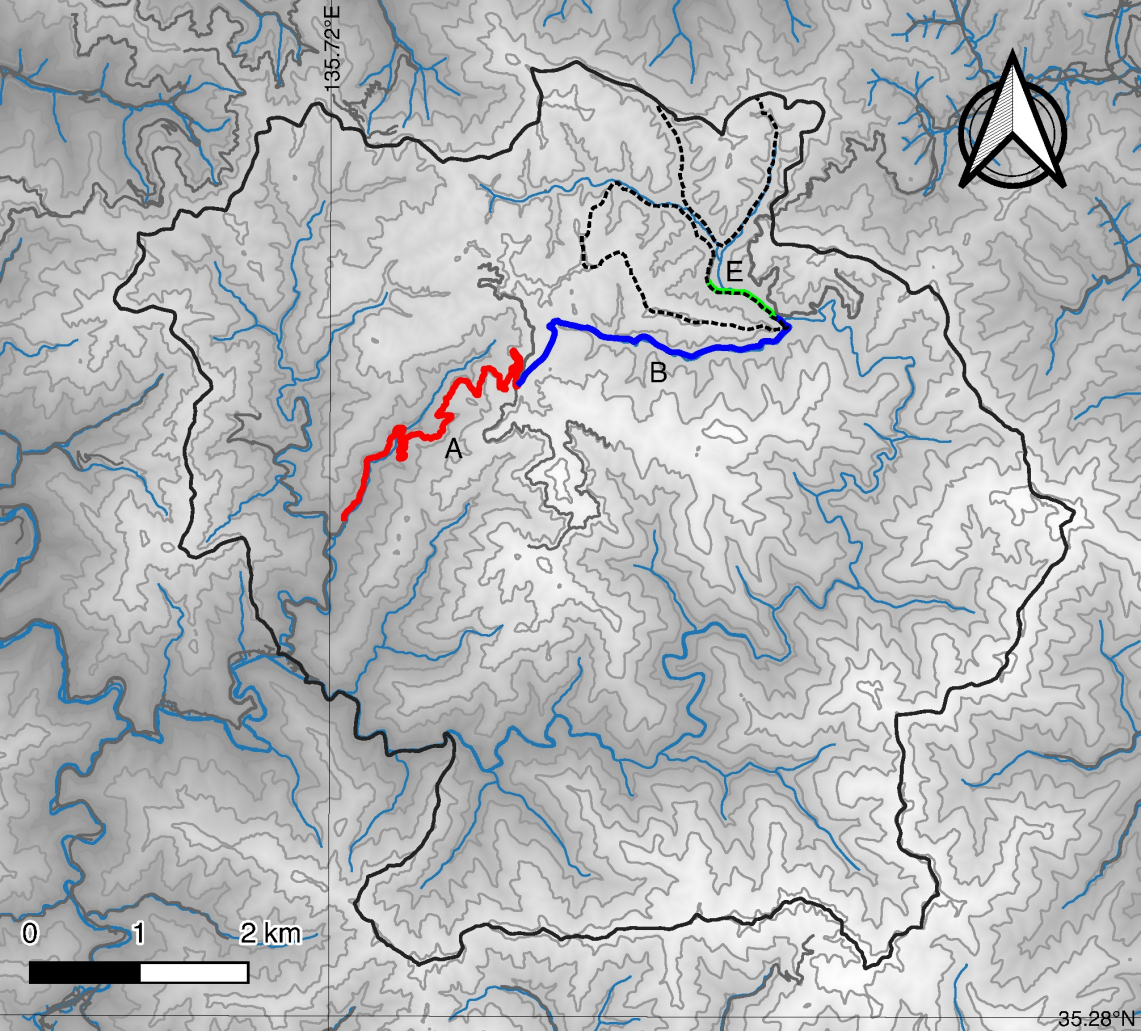
Topographic map of Ashiu Forest Station and the surrounding area. Red, blue, and green lines denote the location of the selected route sectors, A, B, and E, respectively. The parts surrounded by solid lines denote the area of Ashiu Forest Station and the area surrounding. The parts surrounded by broken lines denote survey area by block count.

The study site was located at the Ashiu Forest Research Station, Field Science Education and Research Center, Kyoto University, Japan (35°20′N, 135°45′E; 355–959 m a.s.l., 41.86 km^2^) and the surrounding area (46.12 km^2^ in total, Fig 1). The mean annual temperature and precipitation in this area are 13.1 C and 2,333 mm, respectively [22]. The maximum snowfall during each winter at 356 m elevation was 31.0–141.7 cm between 2007 and 2018 (Table 1). The forest is usually closed from January to early April because the roads in the forest must be blocked with snow. This forest is located in the transition part between the temperate deciduous forest zone and the warm temperate forest zone. This area is well known for being highly diverse in plant species and existing phylogeographically important populations of some species in the forest [22]. Though the forest is one of the wildlife protection areas in Japan, forest vegetation has been steadily degraded by the browsing of *C*. *nippon* [23, 24]; thus, nuisance control started in 2008 using guns, traps, and cages (Table 2). The last known Japanese wolf (*Canis lupus hodophilax*) was caught in the Nara prefecture in 1905 and there have been no sightings of it in Japan since. Thus, we considered that potent predators of deer such as wolves had been extinct all over Japan, including in our study site.

**Table 1:** The numbers of deer carcasses in winter and winter climate.

Year	Carcasses^a^	Distance (km)^b^	SD50^c^ (days)	MaxS^d^ (cm)
Nov 2007—Mar 2008	30	78.5	52	132
Nov 2008—Mar 2009	0	85.0	40	130
Nov 2009—Mar 2010	0	98.5	0	36.7
Nov 2010—Mar 2011	25	88.5	76	141.7
Nov 2011—Mar 2012	4	83.5	87	138.3
Nov 2012—Mar 2013	7	101.5	21	90.7
Nov 2013—Mar 2014	6	83.5	70	114
Nov 2014—Mar 2015	38	100.0	71	113
Nov 2015—Mar 2016	2	92.5	0	31
Nov 2016—Mar 2017	30	86.5	61	135
Nov 2017—Mar 2018	0	93.0	7	87

^a^Number of deer carcasses

^b^Distance of the total survey area during spring thaw

^c^Snow cover >50 cm duration

^d^Maximum snow depth at 356 m elevation at the Ashiu Forest Station.

**Table 2:** The numbers of deer hunted by nuisance control.

	May-June	July-Aug.	Sep.-Oct.	Nov.-Dec.	Total
2007	0	0	0	0	0
2008	0	0	0(6)	10(24)	10(30)
2009	0(6)	0	0(6)	3(20)	3(32)
2010	0	0	0	7(6)	7(6)
2011	0	0	4(8)	1(8)	5(16)
2012	0	0	5(13)	3(6)	8(19)
2013	15(25)	0	3(14)	4(11)	22(50)
2014	17(12)	1(2)	4(8)	10(4)	32(26)
2015	6(14)	2(4)	2(19)	2(6)	12(43)
2016	8(19)	2(6)	2(10)	5(16)	17(51)
2017	1(14)	0	0	4(20)	5(34)
2018	4(4)	0	2(20)	0	6(24)

In parentheses indicates hunting effort (the product of the number of hunters and days for hunting).

### Road count

We selected three route sectors (A: 4.7 km, B: 3.3 km, E: 0.7 km; Fig 1) to record the numbers of deer sighted. The investigators of this study were mainly researchers and technical staff employed by the Forest Research Station, including non-specialists in deer. They recorded the date, weather, sector name, and time when they began driving through each sector, whenever they drove through a whole sector by vehicle during the period from May 1, 2007 to December 31, 2018. Then, they recorded the number of deer in each sector. If they found no deer, they recorded the number as zero. The details of the survey are described in a previous study [19].

We excluded records that lacked information about the number of deer sighted, sector name, year, and date. We also excluded records from January to April because few records were available from these periods due to snow accumulation and driving speed was different from other seasons. Furthermore, data within 15 minutes before and after were excluded from later analysis because data independence could not be guaranteed. After this data cleaning, we used 8,616 records for later analysis.

### Block count

Block counts were conducted in two sites (north: 86.9 ha, south: 111.7 ha) of the Ashiu Forest Research Station in December, from 2001 to 2018 except for 2017 (Fig 1). The sites were divided into 14 and 19 blocks (5–7 ha per block depending on the terrain), respectively. Each block was thoroughly surveyed by an observer walking in a zig-zag motion along the terrain in order to guarantee good visibility. When an observer spotted deer, they informed the observers of adjacent blocks using transceivers to avoid duplicate counting. Occasionally, we did not survey some blocks due to sudden snowfall and lack of observers; however, the total surveyed area was 181.27 ha in most years. Because it was a missing value only in 2017 and values did not change so much in the previous (2016) and next year (2018), we used the mean of 2016 and 2018 as the value of 2017 in the model described later.

### Number of deer carcasses at spring thaw

We counted the number of deer carcasses found in forest during the thawing period from April to early July, for the years 2005–2018. We needed to find deer carcasses emerging from the snow before animals preyed on them. However, we could not distinguish their age and sex because the parts of carcasses bodies were sometimes scattered around. We covered a 1–21 km distance per survey and repeated the procedure for 10–18 times per year to look for deer carcasses across the forest (Table 1 and S1 Fig). In addition to looking for dead deer, we also relied on our sense of smell and detected carcasses based on the odor they emitted.

### State–space model

We analyzed field observation data of relative abundance indices of deer with state–space models, based on a hierarchical Bayesian framework [10, 25, 26]. The state–space model divided the observation data into a system model, representing “true” but unknown population size, and an observation model that accounts for error in counts caused by ability of observers [25, 26]. Because most observers were not specialists for animals, they sometimes missed the count. The state–space models allowed us to permit potential errors in the count data. In most past studies in deer population dynamics, the analysis was performed on a yearly basis. However, we set the time interval to 2 months, excluding the period from January to April (*t* = 1 in May and June 2007, *t* = 2 in July and August 2007, *t* = 3 in September and October 2007, *t* = 4 in November and December 2007, *t* = 5 in May and June 2008, etc.). This was because we were able to use the road count data from all year round except from January to April (when the forest was covered by snow). We wanted to know the seasonal in addition to the annual fluctuation.

#### System models

Expected deer abundance at time *t* (*N*_*t*_) in the forest depended on expected deer abundance at time *t* −1 (*N*_*t*−1_); the number varied with the effect of population growth (*r*_*t*_) including birth, natural mortality, immigration, migration at time *t* (*r*_*t*_ did not include the effects of hunting and mortality due to snowfall), and the effect of hunting at time *t* (hunting rate: *h*_*t*_). It can be expressed as follows:

$$N_{t}=N_{t}{}_{–1}\times r_{t}\times \left(1-h_{t}\right)$$ (1)

During the season when the forest was covered by snow (January to April), the deer sometimes got stuck or starved, due to lack of food as a result of the heavy snow. We defined the mortality rate during the seasons when the forest was covered by snow, just before the time *t*, as *d*_*t*_. Then, *N*_*t*_ (*t* = 5, 9, 13, …45) can be expressed as follows:

$$N_{t}=N_{t}{}_{-1}\times r_{t}{}^{2}\times \left(1-h_{t}\right)\times \left(1-d_{t}\right)$$ (2)

Although we set the time interval to two months during May to December, we set it to 4 months during January to April. Thus we squared *r*_*t*_ in (2).

If we calculate the logarithm of the two aforementioned equations, then the process follows a linear structure. Then, Eqs (1) and (2) can be re-written as follows:

$$NL_{t}=NL_{t}{}_{-1}+rl_{t}+\log\left(1-h_{t}\right)\;\left(t=2,3,4,6,7,\ldots ,48\right)$$ (3)

$$NL_{t}=NL_{t}{}_{-1}+2\times rl_{t}+\log\left(1-h_{t}\right)+\log\left(1-d_{t}\right)\;\left(t=5,9,13,\ldots ,45\right)$$ (4)

We introduced stochasticity into the deer population dynamics. Then, Eq (3) can be expressed as follows:

$$NL_{t}∼Normal\left(\mu_{t},\sigma_{1}{}^{2}\right)\;\left(t=2,3,4,6,7,\ldots ,48\right)$$

$$\mu_{t}=NL_{t–}{}_{1}+rl_{t}+\log\left(1-h_{t}\right)$$

Eq (4) can be expressed as follows:

$$NL_{t}∼Normal\left(\mu_{t},\sigma_{2}{}^{2}\right)\;\left(t=5,9,13,\ldots ,45\right)$$

$$\mu_{t}=NL_{t–}{}_{1}+2\times rl_{t}+\log\left(1-h_{t}\right)+\log\left(1-d_{t}\right)$$

For the time interval we skipped four months every eight months, because we did not use the data collected from road count surveys by vehicles from the winter season (January to April). Thus, we defined different standard deviations of posterior distribution for deer abundance in the logarithmic scale (*σ*_1_ and *σ*_2_).

The prior probability distribution of the log of expected deer abundance in the first year (*NL*_*1*_) was determined as follows:

$$NL_{1}∼Normal\left(0,100^{2}\right)$$

Because time interval was short, the population growth rate (logarithmic scale) at time *t* (*rl*_*t*_) depended on those at time *t* -1 (*rl*_*t* − 1_). Thus it modeled as follows:

$$rl_{t}∼Normal(rl_{t-1},\;\sigma_{3}{}^{2})$$

We did not include a density-dependence parameter in the population growth rate. The density dependence in population growth of sika deer within only 25.3 km^2^ in open ecosystem (4,465 km^2^) was found [27]. However, it was largely depended on the habitat environment. Because our study sites were small (46.12 km^2^), we did not consider habitat heterogeneity in the model. Therefore, we did not consider the density-dependence parameter in this study.

The hunting rate (*h*_*t*_) was the inverse logit transform of the hunting rate in logit scale (*hl*_*t*_). *hl*_*t*_ modeled as follows:

$$hl_{t}∼Normal(\varphi ,\sigma_{4}{}^{2})$$

$$\varphi =hm+rho\times Ef_{t}$$

where *φ* is the mean hunting rate (logit scale) and *σ*_4_ is the standard deviation of posterior distribution of the logit hunting rate, and *hm* is the hunting rate at the forest. Because hunting rate was assumed to increase when the hunting effort (*Ef*_*t*_: the product of the number of hunters and days for hunting in time *t*) increases, we considered the effect of hunting effort on hunting rate in logit scale (*rho*).

The prior probability distribution of *hm* and *rho* was determined as follows:

$$hm∼Normal\left(0,\;100^{2}\right)$$

$$rho∼Normal(0,\;100^{2})$$

The mortality rate during the seasons when the forest was covered by snow (*d*_*t*_) was the inverse logit transform of the mortality rate in logit scale (*dl*_*t*_). *dl*_*t*_ was modeled as follows:

$$dl_{t}∼Normal\left(\varepsilon_{t},\sigma_{5}{}^{2}\right)\;\left(t=5,9,13,\ldots ,45\right)$$

$$\varepsilon_{t}=b+a\times Sn_{t}$$

Where *ε*_*t*_ is the mean mortality during the winter with severe snowfall in time *t* at logit scale and *σ*_5_ is the standard deviation of the posterior distribution of mortality during the winter with snowfall, in the logit scale. Because the mortality may increase in severe snowfall conditions, it was assumed to increase linearly with the number of days with a snow depth of > 50 cm (*Sn*) before time *t*. To consider the different effects of snowfall, we also used the maximum snow depth instead of the number of days with snow depth of > 50 cm (S2 Table). The *b* and *a* were the intercept and coefficient, respectively. The prior probability distributions of *b* and *a* were as follows:

$$b∼Normal\left(0,\;100^{2}\right)$$

$$a∼Normal\left(0,\;100^{2}\right)$$

We assigned weakly informative priors for scale parameters, σ_1_ to σ_5_ as *Cauchy*(0, 10).

#### Observation models

We modeled the number of deer seen in road count surveys (*C*_*t*,*m*_) in time *t* in route *m* (*m* = a, b, e) as follows:

$$C_{t,m}∼Poisson(\delta_{t,m})$$

$$\delta_{t,m}=N_{t}\times R_{m}\times rS_{t}\times O_{t,m}\times A_{c,m}$$

where *R*_*m*_ is the observation rate per survey in route *m* that converts *N*_*t*_ to *δ*_*t*,*m*_, *rS*_*t*_ is the seasonal observation rate per survey in time *t*, *O*_*t*,*m*_ is the number of survey occasions conducted over two months for each route, and *A*_*c*,*m*_ is the ratio of the study area in each drive count route (we assumed the census width to be 15m) per that of forest (a: 0.153%, b:0.107%, c: 0.023%). We assumed *C*_*t*,*m*_ followed a Poisson distribution. More precisely, the probability distribution of *C*_*t*,*m*_ is a Poisson/log-normal mixture because *N*_*t*_ is assumed to follow a log-normal distribution. Ideally, *C*_*t*,*m*_ should be modeled to follow a binomial distribution with the population size and the observation rate for each route and time. However, replicated measurements are typically required to estimate these parameters explicitly [28]. Unfortunately, our data did not have such a structure, so we only estimated the expected population size in this model. This was the same for *B*_*t*_, *H*_*t*_, and *D*_*t*_, mentioned below. The prior probability distribution of *R*_*m*_ were as follows:

$$R_{m}∼Uniform\left(0,\;1\right)$$

The seasonal observation rate (*rS*_*t*_) was the inverse logit transform of the seasonal observation rate in logit scale (*rsl*_*t*_). *rsl*_*t*_ would be affected by leaf phenology of understory vegetation and braches of trees and deer activity including their lactation, mating and so on. The fluctuation of this seasonality in observation rate would have periodicity and the sum of them would be small [29]. Thus, we modeled these seasonal effects as follows;

$$rsl_{t}=-\sum_{\left(l=1\right)}^{3}rsl_{\left(t-l\right)}+\omega t\;(t=4,5,6,\ldots ,48)$$

$$\omega_{t}∼Normal(0,\;\sigma_{6}{}^{2})$$

where *ω*_*t*_ is the noise term and *σ*_6_ is the standard deviation of posterior distribution of *ω*_*t*_. We assigned weakly informative priors for scale parameters, σ_6_ as *Cauchy*(0, 10).

We modeled the number of deer seen by block count (*B*_*t*_) as follows:

$$B_{t}∼Poisson\left(\theta_{t}\right)\;\left(t=5,9,13,\ldots ,45\right)$$

$$\theta_{t}=N_{t}\times bc\times A_{b,t}$$

where *θ*_*t*_ is the mean number of deer seen by block count in time *t*, *bc* is the observation rate per unit area that converts *N*_*t*_ to *θ*_*t*_, and *A*_*b*,*t*_ is the survey area of the block count in time *t*. We assumed *B*_*t*_ followed a Poisson distribution, although Poisson/log-normal mixture is more accurate description we have already mentioned. The prior probability distribution of *bc* was as follows:

bc∼Uniform(0,1)

We modeled the number of deer hunted by nuisance control (*H*_*t*_) as follows:

$$H_{t}∼Poisson\left(\lambda_{t}\right)\;\left(t=2,3,4,\ldots ,48\right)$$

$$\lambda_{t}=N_{t}\times h_{t}$$

where *λ*_*t*_ is the mean number of deer hunted in time *t*. We assumed *H*_*t*_ followed a Poisson distribution, although Poisson/log-normal mixture is more accurate description we have already mentioned.

We modeled the number of deer carcasses found after thawing (*D*_*t*_) as follows:

$$D_{t}∼Poisson\left(\eta_{t}\right)\;\left(t=5,9,13,\cdots ,45\right)$$

$$\eta_{t}=N_{t}\times d_{t}\times rD\times A_{d,t}$$

where *η*_*t*_ is the number of dead deer found after thawing in time *t*, *rD* is the detection rate per unit area that converts *N*_*t*_ to *η*_*t*_, and *A*_*d*,*t*_ is the survey area of dead deer surveyed after thawing in time *t*. We assumed *D*_*t*_ followed a Poisson distribution, although Poisson/log-normal mixture is more accurate description we have already mentioned. The parameter estimation was performed by the Markov Chain Monte Carlo (MCMC; [30]) calculation using RStan 2.18.2 [31]. We ran four parallel MCMC chains and retained 60,000 iterations after an initial burn-in of 30,000 iterations. We thinned sampled values to 1.0%. Convergence of MCMC sampling was judged by the criterion that the potential scale reduction factor on split chains, $\hat{R}$ was smaller than 1.1 [32] and by a check of the MCMC trace.

The predicted total deer abundance for each time was drawn from a Poisson distribution with the mean as *N*_*t*_. To evaluate our models, we compared observed data to simulated data from the posterior predictive distribution [33]. We generated 1,000 data used for posterior predictive checks which we simulate from the posterior predictive distribution.

## Results

The $\hat{R}$ values of our estimated parameters were all under 1.1. The estimated deer abundance had a sharp peak (September–October 2010, Fig 2) during the 12-year period. From 2011 to 2018, the estimated deer abundance was stable compared to the other periods. The models indicated seasonal patterns in deer abundance; deer abundance gradually increased from April to December during 2013 and 2018. The mean of observation rates in route E was higher than that in routes A and B (Table 3). The 95% credible interval (CI) of *hm* and *rho* was −8.20 to −6.80 and 0.01 to 0.15, respectively. On the other hand, the 95% CI of *a* included 0. Even when maximum snow depth was used instead of the number of days with snow depth of > 50 cm, the 95% CI of *a* included 0 (S3 Table).

**Fig 2:**
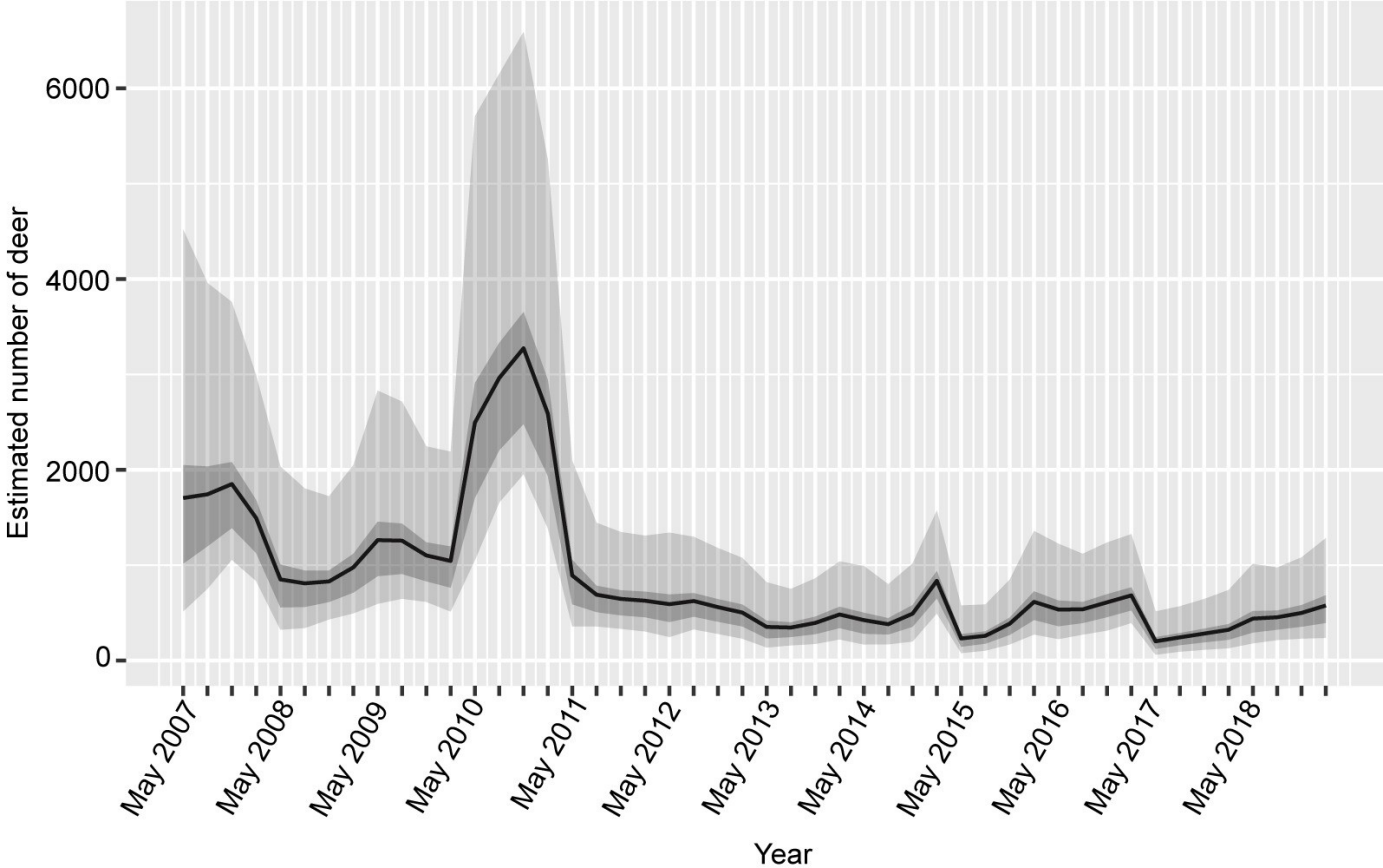
Estimated deer abundances that were obtained from the state–space model from 2007 to 2018. The black line denotes the mean of estimated deer abundance. The 50% and 95% credible intervals are denoted the dark and light gray, respectively.

**Table 3:** Data and parameters designed to estimate deer abundances from multiple abundance indices and posterior summaries of coefficients from the model.

Parameter	Definition	Mean	Lower bound of 95% CI	Upper bound of 95% CI
System model				
*N*_*t*_	expected deer abundance in time *t*			
*NL*_*t*_	log(*N*_*t*_)			
*μ*_*t*_	mean of *logN*_*t*_			
*r*_*t*_	population growth rate in time *t*			
*rl*_*t*_	log(*r*_*t*_)			
*d*_*t*_	winter mortality in time *t*			
*dl*	logit of winter mortality			
*h*_*t*_	hunting rate in time *t*			
*hl*	logit of hunting rate			
*φ*	mean of *hl*			
*hm*	logit of hunting rate at the forest	−6.06	−7.21	−5.06
*Ef*_*t*_	hunting effort (the product of the number of hunters and days for hunting in time *t*)			
*rho*	effect of hunting effort on hunting rate in logit scale	0.08	0.01	0.15
*ε*_*t*_	mean of *dl*			
*b*	intercept of snow effect on the winter mortality	−4.97	−10.30	−1.30
*a*	coefficient of snow effect on the winter mortality	0.06	−0.01	0.15
*Sn*	numbers of days with snow depth of > 50 cm			
*σ*_1_	Scale parameter of a Normal distribution that is a prior of *μ*_*t*_ at *t* = 2,3,4,6,7, …, 48	0.29	0.04	0.59
*σ*_2_	Scale parameter of a Normal distribution that is a prior of *μ*_*t*_ at *t* = 5,9,13, …., 45	0.69	0.14	1.43
*σ*_3_	Scale parameter of a Normal distribution that is a prior of *rl*_*t*_	0.06	0.01	0.17
*σ*_4_	Scale parameter of a Normal distribution that is a prior of *hl*	0.99	0.62	1.51
*σ*_5_	Scale parameter of a Normal distribution that is a prior of *ε*_*t*_	3.03	1.42	6.37
*σ*_6_	Scale parameter of a Normal distribution that is a prior of *ω*_*t*_	0.77	0.40	1.29
Observation model				
*C*_*t*,*m*_	number of deer seen in time *t* in route *m* by road count surveys			
*δ*_*t*,*m*_	mean of *C*_*t*,*m*_			
*rsl*_*t*_	seasonal observation rate in logit scale in time *t*			
*rS*_*t*_	inverse logit of *rsl*_*t*_			
*ω*_*t*_	noise term of *rsl*_*t*_			
*O*_*t*,*m*_	number of road count survey occasions during two months in time *t* in route *m*			
*A*_*c*,*m*_	ratio of surveyed area by road count surveys in route *m* per forest area			
*R*_*a*_	observation rate at drive count at route A	0.10	0.05	0.15
*R*_*b*_	observation rate at drive count at route B	0.13	0.06	0.20
*R*_*e*_	observation rate at drive count at route E	0.75	0.35	0.99
*B*_*t*_	number of deer seen in time *t* by block count surveys (*t* = 5,9,13, …,45)			
*θ*_*t*_	mean of *B*_*t*_			
*bc*	observation rate at block count	0.21	0.06	0.31
*Area*_*b*,*t*_	ratio of surveyed area by block count per forest area in time *t*			
*H*_*t*_	number of hunted deer in time t by nuisance control			
*λ*_*t*_	mean of *H*_*t*_			
*D*_*t*_	number of deer carcasses in time *t* (*t* = 5,9,13, …,45)			
*η*_*t*_	mean of *D*_*t*_			
*rD*	detection rate at deer carcasses survey	0.77	0.40	0.99
*A*_*d*,*t*_	ratio of surveyed area by deer carcasses survey after thawing per forest area in time *t*			

**Fig 3:**
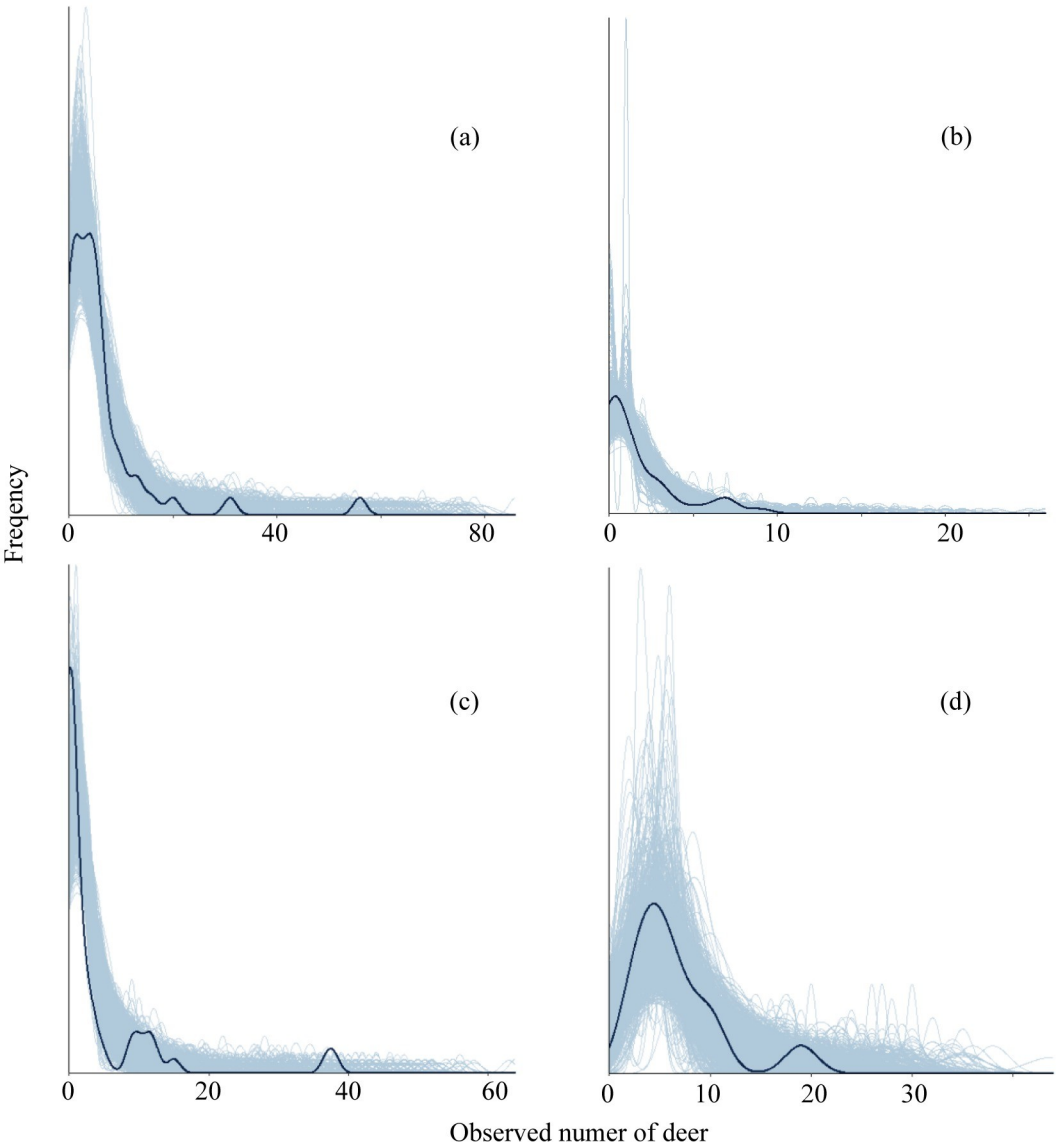
Kernel density estimate of the observed data set (dark blue lines) with density estimates for 1000 simulated data sets drawn from the posterior predictive distribution (light blue lines). (a) *C*_*a*_, (b) *C*_*b*_, (c) *C*_*e*_, (d) B.

**Fig 4:**
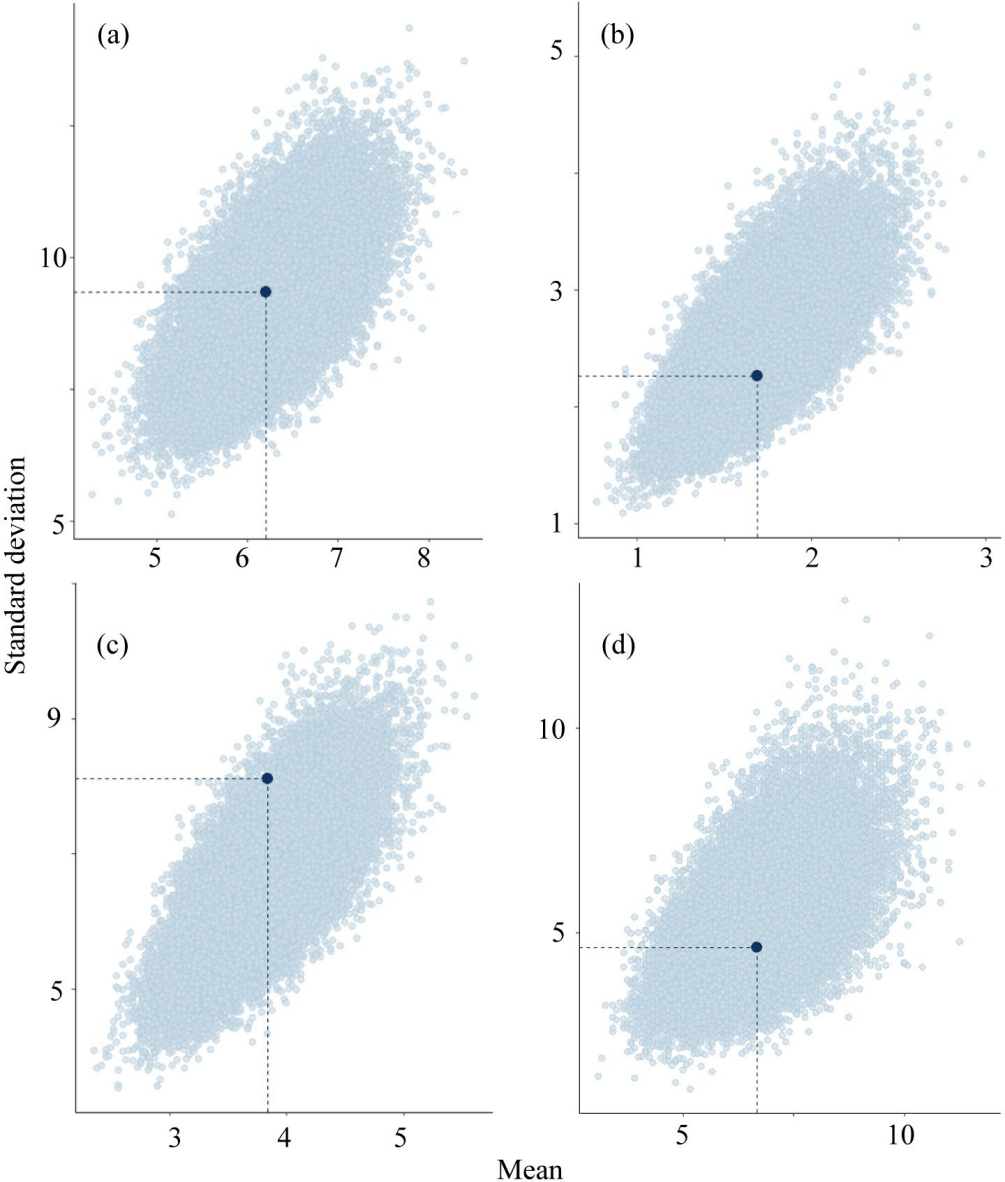
Mean and standard deviation of value of the test statistic computed from the observed values (dark blue dot) and those from the estimated values for 1000 simulated data sets drawn from the posterior predictive distribution (light blue dots). (a) *C*_*a*_, (b) *C*_*b*_, (c) *C*_*e*_, (d) B.

The models was able to simulate new data that was similar to the observed values of number of deer seen in road count surveys in route A and B (*C*_*a*_, *C*_*b*_, respectively) and number of deer seen by block count (*B*) (Fig 3 and Fig 4). Compared to them, models were less able to simulate new data that are similar to the observed value of number of deer seen in road count surveys in route C (*C*_*e*_).

## Discussion

Using a Bayesian state–space model, we were able to estimate annual and seasonal fluctuations of deer abundance with data collected from block count surveys, road count surveys by vehicles, mortality surveys during the winter, and nuisance control. The models was able to simulate new data that was similar to the observed values of *C*_*a*_, *C*_*b*_, and B, though they were less able to simulate new data that was similar to the observed value of *C*_*e*_ (Fig 3 and Fig 4). It suggested that our model was evaluated as good fit. However, we did not measure detectability of each survey though the distinction of abundance and detectability is very important in the estimation of wildlife abundance [34]. Therefore, we need to adopt the robust design [35] to estimate detection probability in future study. However, to improve the accuracy of the estimation, we tried to combine the multiple surveys because the uncertainty can be mitigated by using multiple indicators [10, 36, 37].

We found a sharp peak of deer abundance during the 12-year study period (September–October, 2010, Fig 2). The estimated deer abundance at the autumn of 2010, in particular, was the highest (71.0 individuals per km^2^). The peak in 2010 could be considered an outbreak; this is also reported in other populations and deer species [17, 38]. In 2011, the estimated deer abundance decreased drastically, and has remained at a low level since then. By 2003, most shrubs, herbs, and dwarf bamboo in the forest had already been overgrazed [23, 39, 40]. Therefore, the 2010 irruption and the 2011 decrease could not be due to food shortage of the understory vegetation. When the understory vegetation was poor, deer may have been depending strongly on nuts from canopy and sub-canopy species as food sources during the autumn. In the autumn of 2009, nut production was synchronously very high in three dominant masting Fagaceae species (*Fagus crenata*, *Quercus crispula* and *Quercus serrata*) in the Hyogo prefecture that lies next to the prefecture the study forest belongs to; then, nut production was synchronously very low in the autumn of 2010 [41]. Although we did not collect any masting data from our study site, a nut shortage may have affected the drastic deer population decrease of 2011. From 2011, the aforementioned three tree species did not produce nuts synchronously. This asynchronous nut production might have led to low deer population stability starting from 2011 onwards. The carrying capacity of deer might change not only spatial heterogeneity of habitat (the ratio of grassland, deciduous forest, and evergreen forest), which was reported in [27], but also temporal heterogeneity of habitats.

The estimated deer abundance was 4.4 to 71.0 individuals per km^2^ in this study. It is within the range of the estimated carrying capacity of sika deer (1.34 to 98.4 individuals per km^2^) in Yamanashi Prefecture in central Japan [27]. Even in the open ecosystems, they found density dependent decline in the population growth rate. Therefore, in 2010, the density dependence in the population growth rate might occur in the study forest. We also need to consider the density-dependence in the population growth rate based on the habitat environment in future.

In this study, the seasonal fluctuation of deer abundance was obscure. It is a little bit different from the past results obtained from road count surveys by vehicles [42]. In the model, we considered the seasonal observation rate. It would be affected by leaf phenology of understory vegetation and braches of trees and deer activity including their lactation, mating and so on. It would purge the apparent seasonal fluctuation. However, the seasonal fluctuation of deer abundance gradually increased from April to December during 2013 and 2018. Though some deer exist in forests even during the winter [40], they migrate seasonally to avoid snow accumulation in heavy snow-covered areas [43, 44]. Therefore, the seasonal variation we detected may be due to the seasonal migration pattern in addition to the population recruitment through fawn births in early summer. The potential browsing pressure increase in the plant community during the summer may have negative effects on herbaceous plants, especially the one that grow in the summer and flowered in the autumn. In this area, as the plants that flower after midsummer are herbaceous and are more severely browsed compared to trees [45], the fitness of pollinators working from summer to autumn may critically decrease due to a shortage in their flower resources.

The 95% credible interval (CI) of *hm* and *rho* ranged from −8.20 to −6.80 and 0.01 to 0.15, respectively (Table 3). These results suggest that nuisance control could be useful in decreasing deer populations and are similar to past results [10]. On the other hand, a previous study [10] pointed out the difficulties of increasing hunting pressures because Japanese hunters were getting older. To establish an effective deer abundance management program under this circumstance, the development of simple and inexpensive capture methods is urgent.

Late snowfall substantially affects the mortality of *C*. *nippon* [12]. In *Cervus elaphus* in Norway, winter harshness affects first-year survival but not the survival of adults [46]. In this study, the 95% CI of *a* included 0. This suggests that snowfall may have slightly affected deer mortality during the winter in the present study. This is similar to results obtained from studying the alpine ungulate *Rupicapra rupicapra* [15], though their population dynamics are largely affected by summer temperature. At first glance, our results seem to suggest that the mortality rate during the winter will not change even if snowfall decreases due to global warming. However, as we mentioned earlier, deer inhabiting regions with heavy snowfall, migrate to safe areas during the winter and go back to their initial habitats after snowmelt. Thus, snowfall decrease due to global warming may decelerate the winter migration of deer and, subsequently, deer that remain on-site may intensively forage evergreen perennial plants during the winter season.

In route E, the observation rate was higher than that in routes A and B (Table 3). In this study, we did not consider the spatial pattern of deer. While route A is close to a village, route E is remote and located deep in montane forest. Therefore, human activity may have affected the observation rate. The topographic pattern could have affected route visibility, though we uniform ranges of observation 15 m width in all routes. Landscape characteristics such as evergreen forests and artificial grasslands affect deer abundance in local areas [10]. As shown in Fig 1, this study site consists of steep slopes and deep valleys. The differences in observation rates among routes may also be due to the differences in landscape characteristics in a local scale in the forest. However, our model did not fit well in road counts at route E. We need to treat the results carefully.

In conclusion, we clarified the population dynamics of deer not only annually but also seasonally. Snowfall accumulations did not affect population dynamics of deer in this study irrespective of higher mortality of deer during the winter [9, 12]. However, we need to pay attention to the effect the winter migration of deer has on plant communities because many deer migrated to another area during the winter and came back before the summer. Although we could not grasp the population dynamics during the snow accumulation season, in warmer winters, more deer may remain in the forest. Thus, a warmer winter may lead to degradation of evergreen perennial plant communities during the winter and early spring. Additional investigation on evergreen perennial plants could help examine the effect of deer browsing during the winter. In contrast to snowfall accumulations, nuisance control had an effect on the population dynamics of deer. Even in wildlife protection areas and national parks where hunting is regulated, nuisance control could be effective in buffering the effects of excessive deer browsing on forest ecosystems as well as plant communities, under the absence of potent predators.

## Supporting information

S1 Fig Survey route where we counted the number of deer carcasses found in forest during the thawing period from April to early July, for the years 2017–2018. The parts surrounded by solid lines denote the area of Ashiu Forest Station and the area surrounding. The red lines denote the survey route of the year. The routes are slightly different from year to year.
(TIF)

S2 Fig Seasonal observation rate (*rS*_*t*_) that were obtained from the state–space model from 2008 to 2018. The black line denotes the mean of estimated deer abundance. The 50% and 95% credible intervals are denoted the dark and light gray, respectively.
(TIF)

S1 Table Numbers of deer observed (A, B, E) and numbers of survey (*O*_*a*_, *O*_*b*_, *O*_*e*_) by road count surveys at each sector, respectively. (DOCX)

S2 Table Numbers of deer observed and area by block count surveys. (DOCX)

S3 Table Data and parameters designed to estimate deer abundances from multiple abundance indices and posterior summaries of coefficients from the model which used MaxS instead of SD50 in Table 1 as snow effect. (DOCX)

S1 Appendix R-Code for fitting the Bayesian state-space model. Simulation code using a Bayesian state–space model.
(DOCX)

S1 Dataset (XLSX)
