# Supplementary figures and images for: Seasonal and annual fluctuations of deer populations estimated by a Bayesian state–space model
Source: PLoS One. 2020 Jun 18;15(6):e0225872. doi: 10.1371/journal.pone.0225872 (PMC7302714; doi:10.1371/journal.pone.0225872)

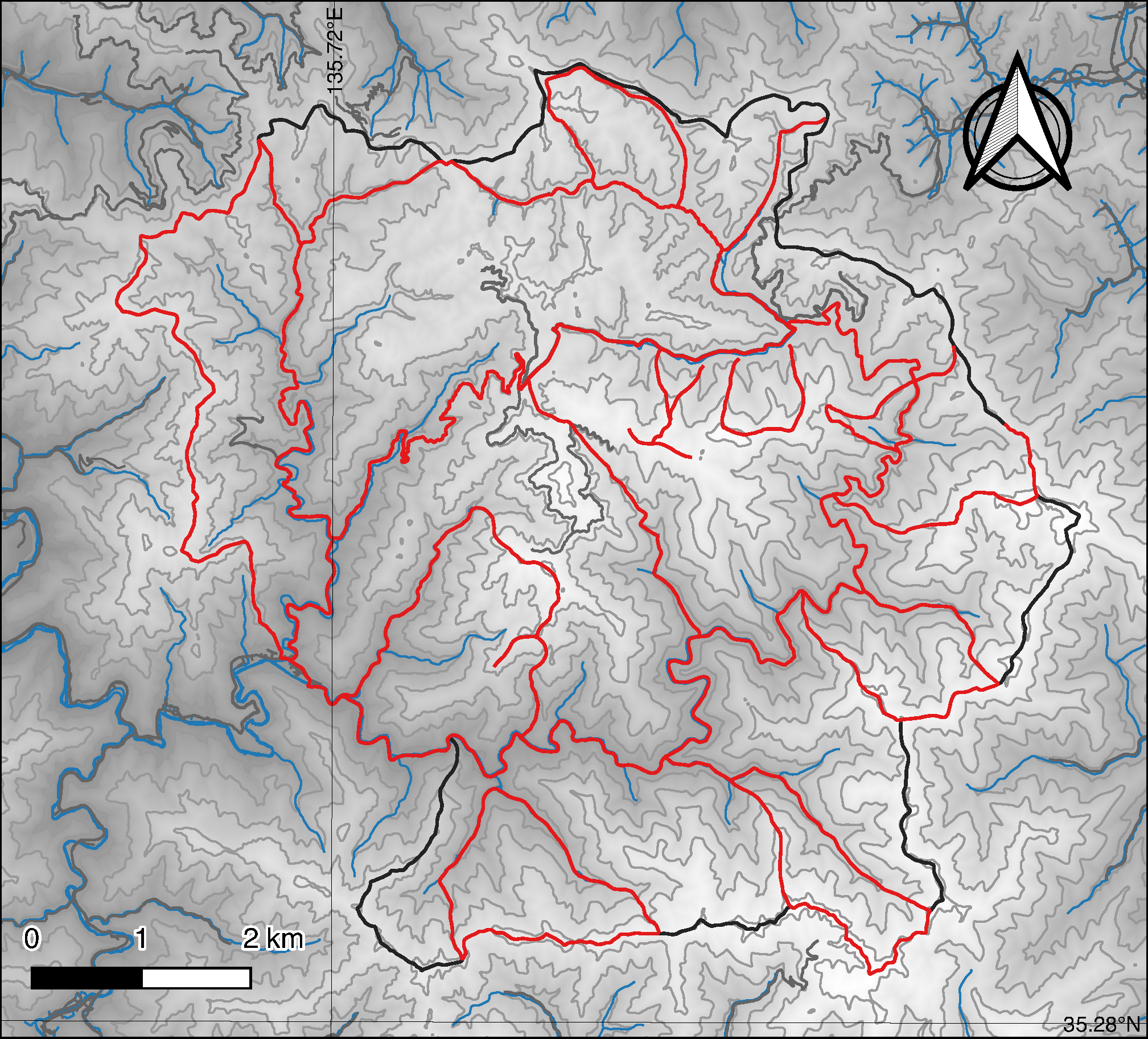

Supplement: S1 Fig — The parts surrounded by solid lines denote the area of Ashiu Forest Station and the area surrounding. The red lines denote the survey route of the year. The routes are slightly different from year to year. (TIF) [file pone.0225872.s001.tif]

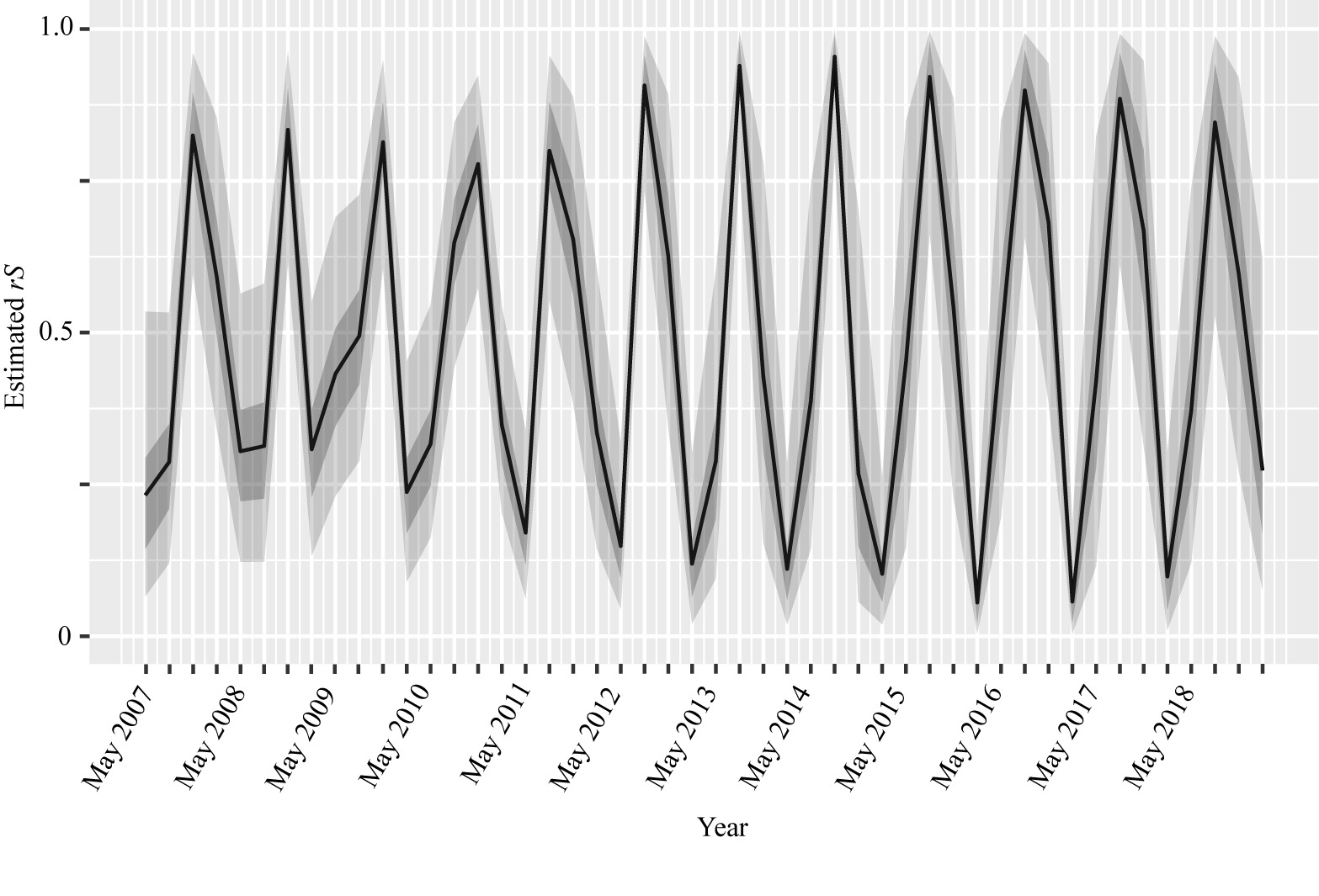

Supplement: S2 Fig — The black line denotes the mean of estimated deer abundance. The 50% and 95% credible intervals are denoted the dark and light gray, respectively. (TIF) [file pone.0225872.s002.tif]
