# Supplementary material for: Seasonal and annual fluctuations of deer populations estimated by a Bayesian state–space model
Source: PLoS One. 2020 Jun 18;15(6):e0225872. doi: 10.1371/journal.pone.0225872 (PMC7302714; doi:10.1371/journal.pone.0225872)
Supplement: S1 Table — (DOCX) [file pone.0225872.s003.docx]

**S1 Table. Numbers of deer observed (A, B, E) and numbers of survey (obA, obB, obC) by road count surveys at each sector, respectively.**

| Year | cycle | A | obA | B | obB | E | obE |
| --- | --- | --- | --- | --- | --- | --- | --- |
| 2007 | 1 | 4 | 61 | 0 | 22 | 1 | 32 |
| 2007 | 2 | 6 | 68 | 0 | 14 | 3 | 50 |
| 2007 | 3 | 31 | 117 | 3 | 19 | 12 | 55 |
| 2007 | 4 | 13 | 55 | 2 | 17 | 0 | 19 |
| 2008 | 5 | 1 | 79 | 3 | 23 | 1 | 26 |
| 2008 | 6 | 3 | 83 | 1 | 28 | 0 | 25 |
| 2008 | 7 | 8 | 94 | 7 | 27 | 1 | 42 |
| 2008 | 8 | 6 | 92 | 0 | 28 | 0 | 30 |
| 2009 | 9 | 13 | 86 | 1 | 46 | 2 | 50 |
| 2009 | 10 | 7 | 102 | 2 | 24 | 11 | 82 |
| 2009 | 11 | 9 | 96 | 7 | 36 | 12 | 76 |
| 2009 | 12 | 4 | 79 | 0 | 20 | 0 | 42 |
| 2010 | 13 | 10 | 106 | 1 | 29 | 9 | 50 |
| 2010 | 14 | 20 | 104 | 4 | 38 | 37 | 77 |
| 2010 | 15 | 56 | 141 | 9 | 36 | 38 | 90 |
| 2010 | 16 | 16 | 103 | 5 | 38 | 5 | 54 |
| 2011 | 17 | 2 | 126 | 0 | 51 | 3 | 80 |
| 2011 | 18 | 6 | 106 | 1 | 34 | 10 | 87 |
| 2011 | 19 | 6 | 123 | 6 | 41 | 2 | 76 |
| 2011 | 20 | 5 | 82 | 0 | 27 | 1 | 45 |
| 2012 | 21 | 2 | 159 | 1 | 52 | 0 | 80 |
| 2012 | 22 | 4 | 106 | 1 | 22 | 15 | 82 |
| 2012 | 23 | 3 | 128 | 0 | 44 | 9 | 71 |
| 2012 | 24 | 0 | 86 | 0 | 23 | 0 | 63 |
| 2013 | 25 | 2 | 139 | 1 | 60 | 0 | 64 |
| 2013 | 26 | 1 | 104 | 3 | 47 | 4 | 44 |
| 2013 | 27 | 4 | 121 | 0 | 32 | 0 | 43 |
| 2013 | 28 | 1 | 76 | 0 | 29 | 0 | 39 |
| 2014 | 29 | 6 | 176 | 0 | 53 | 0 | 49 |
| 2014 | 30 | 6 | 109 | 0 | 35 | 0 | 37 |
| 2014 | 31 | 0 | 145 | 0 | 41 | 1 | 69 |
| 2014 | 32 | 0 | 75 | 0 | 22 | 1 | 46 |
| 2015 | 33 | 0 | 124 | 2 | 42 | 0 | 34 |
| 2015 | 34 | 2 | 91 | 0 | 29 | 0 | 35 |
| 2015 | 35 | 4 | 106 | 1 | 28 | 0 | 36 |
| 2015 | 36 | 0 | 65 | 0 | 22 | 0 | 36 |
| 2016 | 37 | 5 | 114 | 1 | 25 | 0 | 32 |
| 2016 | 38 | 10 | 98 | 1 | 30 | 0 | 41 |
| 2016 | 39 | 4 | 100 | 7 | 38 | 0 | 38 |
| 2016 | 40 | 0 | 58 | 0 | 15 | 0 | 34 |
| 2017 | 41 | 0 | 87 | 0 | 27 | 1 | 29 |
| 2017 | 42 | 1 | 72 | 1 | 29 | 3 | 38 |
| 2017 | 43 | 2 | 61 | 1 | 22 | 0 | 22 |
| 2017 | 44 | 0 | 51 | 0 | 23 | 0 | 24 |
| 2018 | 45 | 2 | 107 | 3 | 48 | 0 | 62 |
| 2018 | 46 | 4 | 89 | 2 | 31 | 2 | 33 |
| 2018 | 47 | 5 | 112 | 3 | 32 | 0 | 36 |
| 2018 | 48 | 4 | 75 | 1 | 35 | 0 | 40 |
