# Supplementary material for: Seasonal and annual fluctuations of deer populations estimated by a Bayesian state–space model
Source: PLoS One. 2020 Jun 18;15(6):e0225872. doi: 10.1371/journal.pone.0225872 (PMC7302714; doi:10.1371/journal.pone.0225872)
Supplement: S2 Table — (DOCX) [file pone.0225872.s004.docx]

**S2 Table. Numbers of deer observed and area by block count surveys.**

| Year | Number | Area (ha) |
| --- | --- | --- |
| 2007 | 5 | 181.5 |
| 2008 | 10 | 181.5 |
| 2009 | 7 | 181.5 |
| 2010 | 19 | 181.5 |
| 2011 | 4 | 181.5 |
| 2012 | 2 | 131.2 |
| 2013 | 5 | 181.5 |
| 2014 | 7 | 181.5 |
| 2015 | 10 | 199.4 |
| 2016 | 5 | 199.4 |
| 2017 | - | - |
| 2018 | 3 | 199.4 |
