# Supplementary material for: Seasonal and annual fluctuations of deer populations estimated by a Bayesian state–space model
Source: PLoS One. 2020 Jun 18;15(6):e0225872. doi: 10.1371/journal.pone.0225872 (PMC7302714; doi:10.1371/journal.pone.0225872)
Supplement: S3 Table — (DOCX) [file pone.0225872.s005.docx]

**S3 Table 3. Data and parameters designed to estimate deer abundances from multiple abundance indices and posterior summaries of coefficients from the model which used MaxS instead of SD50 in Table 1 as snow effect.**

| Parameter | | Definition | Mean | Lower bound of 95% CI | Upper bound of 95% CI |
| --- | --- | --- | --- | --- | --- |
| System model | |  |  |  |  |
|  | *N_t_* | Deer abundance in time *t* |  |  |  |
|  | *NL_t_* | log(*N_t_* ) |  |  |  |
|  | *μ_t_* | mean of *logN_t_* |  |  |  |
|  | *r_t_* | population growth rate in time *t* |  |  |  |
|  | *rl_t_* | log(*r_t_*) |  |  |  |
|  | *d_t_* | winter mortality in time *t* |  |  |  |
|  | *dl* | logit of winter mortality |  |  |  |
|  | *h_t_* | hunting rate in time *t* |  |  |  |
|  | *hl* | logit of hunting rate |  |  |  |
|  | *φ* | mean of *hl* |  |  |  |
|  | *hm* | logit of hunting rate at the forest | −6.07 | −7.20 | −5.06 |
|  | *Ef_t_* | hunting effort (the product of the number of hunters and days for hunting in time *t*) |  |  |  |
|  | *rho* | effect of hunting effort on hunting rate in logit scale | 0.08 | 0.01 | 0.15 |
|  | *ε_t_* | mean of *dl* |  |  |  |
|  | *b* | intercept of snow effect on the winter mortality | −6.33 | −14.67 | 0.09 |
|  | *a* | coefficient of snow effect on the winter mortality | 0.04 | −0.02 | 0.11 |
|  | *Sn* | numbers of days with snow depth of > 50 cm |  |  |  |
|  | *σ*_1_ | Scale parameter of a Normal distribution that is a prior of *μ_t_* at *t* = 2,3,4,6,7, …, 48 | 0.31 | 0.15 | 0.60 |
|  | *σ*_2_ | Scale parameter of a Normal distribution that is a prior of *μ_t_* at *t* = 5,9,13, …., 45 | 0.68 | 0.33 | 1.45 |
|  | *σ*_3_ | Scale parameter of a Normal distribution that is a prior of *rl* | 0.05 | 0.04 | 0.16 |
|  | *σ*_4_ | Scale parameter of a Normal distribution that is a prior of *hl* | 0.99 | 0.23 | 1.51 |
|  | *σ*_5_ | Scale parameter of a Normal distribution that is a prior of *ε_t_* | 3.20 | 1.44 | 6.86 |
|  | *σ*_6_ | Scale parameter of a Normal distribution that is a prior of *ω_t_* | 0.78 | 0.23 | 1.30 |
|  |  |  |  |  |  |
| Observation model | | |  |  |  |
|  | *C_t,m_* | number of deer seen in time *t* in route *m* by road count surveys |  |  |  |
|  | *δ_t,m_* | mean of *C_t,m_* |  |  |  |
|  | *rsl_t_* | seasonal observation rate in logit scale in time *t* |  |  |  |
|  | *rS_t_* | inverse logit of *rsl_t_* |  |  |  |
|  | *ω_t_* | sum of *rsl_t_* |  |  |  |
|  | *O_t,m_* | number of road count survey occasions during two months in time *t* in route *m* |  |  |  |
|  | *A_c,m_* | ratio of surveyed area by road count surveys in route *m* per forest area |  |  |  |
|  | *R_a_* | observation rate at drive count at route A | 0.10 | 0.05 | 0.15 |
|  | *R_b_* | observation rate at drive count at route B | 0.13 | 0.06 | 0.20 |
|  | *R_e_* | observation rate at drive count at route E | 0.75 | 0.35 | 0.99 |
|  | *B_t_* | number of deer seen in time *t* by block count surveys (*t* = 5,9,13, …,45) |  |  |  |
|  | *θ_t_* | mean of *B_t_* |  |  |  |
|  | *bc* | observation rate at block count | 0.21 | 0.06 | 0.31 |
|  | *Area_b,t_* | ratio of surveyed area by block count per forest area in time *t* |  |  |  |
|  | *H_t_* | number of hunted deer in time t by nuisance control |  |  |  |
|  | *λ_t_* | mean of *H_t_* |  |  |  |
|  | *D_t_* | number of deer carcasses in time *t* (*t* = 5,9,13, …,45) |  |  |  |
|  | *η_t_* | mean of *D_t_* |  |  |  |
|  | *rD* | detection rate at deer carcasses survey | 0.77 | 0.40 | 0.99 |
|  | *A_d,t_* | ratio of surveyed area by deer carcasses survey after thawing per forest area in time *t* |  |  |  |
