# Supplementary material for: Seasonal and annual fluctuations of deer populations estimated by a Bayesian state–space model
Source: PLoS One. 2020 Jun 18;15(6):e0225872. doi: 10.1371/journal.pone.0225872 (PMC7302714; doi:10.1371/journal.pone.0225872)
Supplement: S1 Appendix — Simulation code using a Bayesian state–space model. (DOCX) [file pone.0225872.s006.docx]

**S1 Appendix. Rstan-Code for fitting the Bayesian state-space model.** Simulation code using a Bayesian state–space model.

**// Structure of the R-code**

**//1) Modeling (deermodel_ppc200310.stan)**

data {

int<lower=0> St; // Number of years x seasons(4)

int<lower=0> Sy; // Number of years

int<lower=0> D[Sy]; // Number of deer carcasses after winter

int<lower=0> H[St]; // Number of hunted deer

int<lower=0> Ca[St]; // Total number of deer in the drive count at A

int<lower=0> Cb[St]; // Total number of deer in the drive count at B

int<lower=0> Ce[St]; // Total number of deer in the drive count at E

int<lower=0> Oa[St]; // Number of observation occasions in the drive count at A

int<lower=0> Ob[St]; // Number of observation occasions in the drive count at B

int<lower=0> Oe[St]; // Number of observation occasions in the drive count at E

int<lower=0> B[Sy]; // Total number of deer in the block count

real<lower=0> Dis[Sy]; // Distance in the deer carcasses survey

real<lower=0> A[Sy]; // Survey area by the block count

int <lower=0> S[Sy]; // numbers of days with snow depth of >50cm

int<lower=0,upper=1> F[St]; // flag to indicate hunting

int<lower=0> Ef[St]; //hunting effort day*person

}

parameters{

vector[St] NL; // log of deer popl.

vector[St] hl; // logit of hunting rate

vector[Sy-1] dl; // logit of death rate

real<lower=0,upper=1> ra; // obs. rate by drive count at A

real<lower=0,upper=1> rb; // obs. rate by drive count at B

real<lower=0,upper=1> re; // obs. rate by drive count at C

vector[St] rsl; // logit of seasonal obs. rate

real<lower=0,upper=1> bc; // obs. rate by block count

vector[St] rl; // log of popl. growth rate

real hm; // log_hunt

real rho; //effect of hunting effort on hunting rate (logit-scale)

real<lower=0> sigma[6]; // scale params.

real b; // intercept of snow effect

real a; // coefficient of snow effect

real<lower=0,upper=1> rD; // obs. rate after thaw

}

transformed parameters {

vector<lower=0,upper=1>[St] h = inv_logit(hl); // hunting rate

vector<lower=0,upper=1>[Sy-1] d = inv_logit(dl); // death rate

vector<lower=0,upper=1>[St] rS = inv_logit(rsl); // seasonal obs. rate

}

model{

// System model

for (t in 2: St) {

rl[t] ~ normal(rl[t-1],sigma[3]);

if (t % 4 == 1)

NL[t] ~ normal(NL[t - 1] + 2*rl[t]+ log(1 - d[(t - 1) / 4])

+ F[t] * log(1 - h[t]), sigma[2]);

else

NL[t] ~ normal(NL[t - 1] + rl[t]

+ F[t] * log(1 - h[t]), sigma[1]);

}

for (t in 1:St)

hl[t]~ normal(hm+ F[t]*rho*Ef[t], sigma[4]);

for(t in 4: St)

rsl[t] ~ normal(-sum(rsl[(t-3):(t-1)]), sigma[6]);

for (y in 1:(Sy - 1))

dl[y] ~ normal(b + a * S[y], sigma[5]);

// Observation model

for (t in 1: St) {

Ca[t] ~ poisson(exp(NL[t]) .* Oa[t] * ra * (4.7*0.015/46.12) * rS[t]);

Cb[t] ~ poisson(exp(NL[t]) .* Ob[t] * rb * (3.3*0.015/46.12) * rS[t]);

Ce[t] ~ poisson(exp(NL[t]) .* Oe[t] * re * (0.7*0.015/46.12) * rS[t]);

}

for (y in 1:(Sy - 1))

D[y] ~ poisson(exp(NL[y * 4]).* d[y]*rD*(Dis[y]*0.04/46.12)); // season = 4, 8, 12,...

for (t in 1:St)

if (F[t] == 1)

H[t] ~ poisson(exp(NL[t]).* h[t]);

for (y in 1:Sy)

B[y] ~ poisson(exp(NL[y * 4]) * (A[y]*0.01 / 46.12) * bc);

// Priors

sigma ~ cauchy(0, 10);

NL[1] ~ normal(0, 100);

hm~normal(0,100);

b ~ normal(0, 100);

a ~ normal(0, 100);

　rho ~ normal(0,100);

}

generated quantities {

int N[St];

int<lower=0> PCa[St];

int<lower=0> PCb[St];

int<lower=0> PCe[St];

int<lower=0> PB[Sy];

for (t in 1:St) {

N[t] = poisson_rng(exp(NL[t]));

PCa[t] = poisson_rng(exp(NL[t]) .* Oa[t] * ra * (4.7 * 0.015 / 46.12) * rS[t]);

PCb[t] = poisson_rng(exp(NL[t]) .* Ob[t] * rb * (3.3 * 0.015 / 46.12) * rS[t]);

PCe[t] = poisson_rng(exp(NL[t]) .* Oe[t] * re * (0.7 * 0.015 / 46.12) * rS[t]);

}

for (y in 1:Sy)

PB[y] = poisson_rng(exp(NL[y * 4]) * (A[y] * 0.01 / 46.12) * bc);

}

**//2) Estimation including the posterior predictive distribution check**

library(readxl)

library(dplyr)

library(rstan)

library(ggplot2)

library(ggfortify)

rstan_options(auto_write=TRUE)

options(mc.cores=parallel::detectCores())

model_file <-"deermodel_ppc200310.stan"

cat(readLines(file(model_file)), sep='\n')

d_file <--"deer19.xlsx"

d_cycle <-read_xlsx(d_file, sheet=2)

d_year <-read_xlsx(d_file, sheet=1)

head(d_cycle)

head(d_year)

data <-list(St = length(d_cycle$cycle),

Sy = length(d_year$Year),

Ca = d_cycle$A,

Cb = d_cycle$B,

Ce = d_cycle$E,

B = d_year$block,

D = d_year$dead,

S = d_year$SD50,

H = d_cycle$hunt,

F = d_cycle$isHunt,

Ef = d_cycle$effort,

Oa = d_cycle$obA,

Ob = d_cycle$obB,

Oe = d_cycle$obE,

Dis = d_year$dDis,

A = d_year$area)

str(data)

fit3 <- stan(model_file, data = data, seed = 12, chains = 4 , iter = 60000, warmup = 30000, thin = 3, control = list(adapt_delta = 0.95, max_treedepth = 15))

print(fit3, par = c("ra", "rb", "re", "bc", "hm","sigma", "rD", "b", "a", “rho”))

rstan::traceplot(fit3, par = c("ra", "rb", "re", "bc", "hm","sigma", "rD", "b", "a", “rho”))

print(fit3, par=c("N"))

print(fit3, par=c("rl"))

print(fit3, par=c("d"))

print(fit3, par=c("h"))

print(fit3, par=c("rS"))

N <- extract(fit3, par = "N")[["N"]]

N.mean <- apply(N, 2, mean)

N.ci <- apply(N, 2, quantile,probs = c(0.025, 0.25, 0.75, 0.975))

d.mean <- data.frame(Year = d_cycle$cycle, Expected = N.mean)

d.ci <- data.frame(Year = d_cycle$cycle,

Lower = N.ci["2.5%", ],

Upper = N.ci["97.5%", ],

Lower2 = N.ci["25%", ],

Upper2 = N.ci["75%", ])

ggplot(d.mean)+

geom_line(aes(x = Year, y = Expected)) +

geom_ribbon(data = d.ci,

aes(x = Year, ymin = Lower, ymax = Upper), alpha = 0.2) +

geom_ribbon(data = d.ci,

aes(x = Year, ymin = Lower2, ymax = Upper2), alpha = 0.3) +

scale_x_continuous(breaks = 1:48)

S <- extract(fit3, par = "rS")[["rS"]]

S.mean <- apply(S, 2, mean)

S.ci <- apply(S, 2, quantile, probs = c(0.025, 0.25, 0.75, 0.975))

ds.mean <- data.frame(Year = d_cycle$cycle, Expected = S.mean)

ds.ci <- data.frame(Year = d_cycle$cycle,

Lower = S.ci["2.5%", ],

Upper = S.ci["97.5%", ],

Lower2 = S.ci["25%", ],

Upper2 = S.ci["75%", ])

ggplot(ds.mean)+

geom_line(aes(x = Year, y = Expected)) +

geom_ribbon(data = ds.ci,

aes(x = Year, ymin = Lower, ymax = Upper), alpha = 0.2) +

geom_ribbon(data = ds.ci,

aes(x = Year, ymin = Lower2, ymax = Upper2), alpha = 0.3) +

scale_x_continuous(breaks = 1:48)

//Posterior Predictive Check

library(bayesplot)

n <- 1000

Ca <- d_cycle$A

PCa <- extract(fit3, pars = "PCa")[["PCa"]]

ppc_ecdf_overlay(Ca, PCa[sample(nrow(PCa), n), ])

ppc_dens_overlay(Ca, PCa[sample(nrow(PCa), n), ])

ppc_stat_2d(Ca, PCa)

Cb <- d_cycle$B

PCb <- extract(fit3, pars = "PCb")[["PCb"]]

ppc_ecdf_overlay(Cb, PCb[sample(nrow(PCb), n), ])

ppc_dens_overlay(Cb, PCb[sample(nrow(PCb), n), ])

ppc_stat_2d(Cb, PCb)

Ce <- d_cycle$E

PCe <- extract(fit3, pars = "PCe")[["PCe"]]

ppc_ecdf_overlay(Ce, PCe[sample(nrow(PCe), n), ])

ppc_dens_overlay(Ce, PCe[sample(nrow(PCe), n), ])

ppc_stat_2d(Ce, PCe)

B <- d_year$block

PB <- extract(fit3, pars = "PB")[["PB"]]

ppc_ecdf_overlay(B, PB[sample(nrow(PB), n), ])

ppc_dens_overlay(B, PB[sample(nrow(PB), n), ])

ppc_stat_2d(B, PB)
